# The Effect of Climate Variability on Gray Whales (*Eschrichtius robustus*) within Their Wintering Areas

**DOI:** 10.1371/journal.pone.0134655

**Published:** 2015-08-26

**Authors:** Christian J. Salvadeo, Alejandro Gómez-Gallardo U., Mauricio Nájera-Caballero, Jorge Urbán-Ramirez, Daniel Lluch-Belda

**Affiliations:** 1 Universidad Autónoma de Baja California Sur, Ap. Post 19-B, La Paz, B.C.S. 23081, México; 2 Centro de Investigaciones Biológicas del Noroeste S.C. Instituto Politécnico Nacional 195, Playa Palo de Santa Rita Sur, La Paz, B.C.S., C.P. 23096, México; 3 Centro Interdisciplinario de Ciencias Marinas-Instituto Politécnico Nacional, Instituto Politécnico Nacional s/n, Playa Palo de Santa Rita, C. P. 23096, La Paz, B.C.S., México; University of California San Diego, UNITED STATES

## Abstract

The environmental conditions of the breeding and feeding grounds of the gray whale (*Eschrichtius robustus*) fluctuates at inter-annual scales in response to regional and basin climate patterns. Thus, the goals of this study were to assess if there are any relationships between summer sea ice on their feeding ground and counts of gray whale mother-calf (MC) pairs at Ojo de Liebre Lagoon (OLL); and if El Niño Southern Oscillation (ENSO) influences the winter distribution of gray whales MC pairs in the three primary breeding lagoons of OLL, San Ignacio Lagoon (SIL) and Santo Domingo Channel north of Bahia Magdalena (SDCh). Maximum February counts of MC pairs were compared with the length of the open-water season at the Bering Sea during the previous year. Then, an ENSO index and sea surface temperature anomalies outside the primary lagoons was compared with the maximum February counts of MC pairs at these lagoons. Results showed that maximum counts of MC pairs in OLL correlates with sea ice conditions in their feeding grounds from the previous feeding season, and this relationship can be attributed to changes in nutritive condition of females. ENSO-related variability influences distribution of MC pairs in the southern area of SDCh during the warm 1998 El Niño and cold 1999 La Niña. This supports the hypothesis that changes in the whales’ distribution related to sea temperature occurs to reduce thermal-stress and optimize energy utilization for newborn whales. Although this last conclusion should be considered in view of the limited data available from all the whales’ wintering locations in all the years considered.

## Introduction

**Fig 1:**
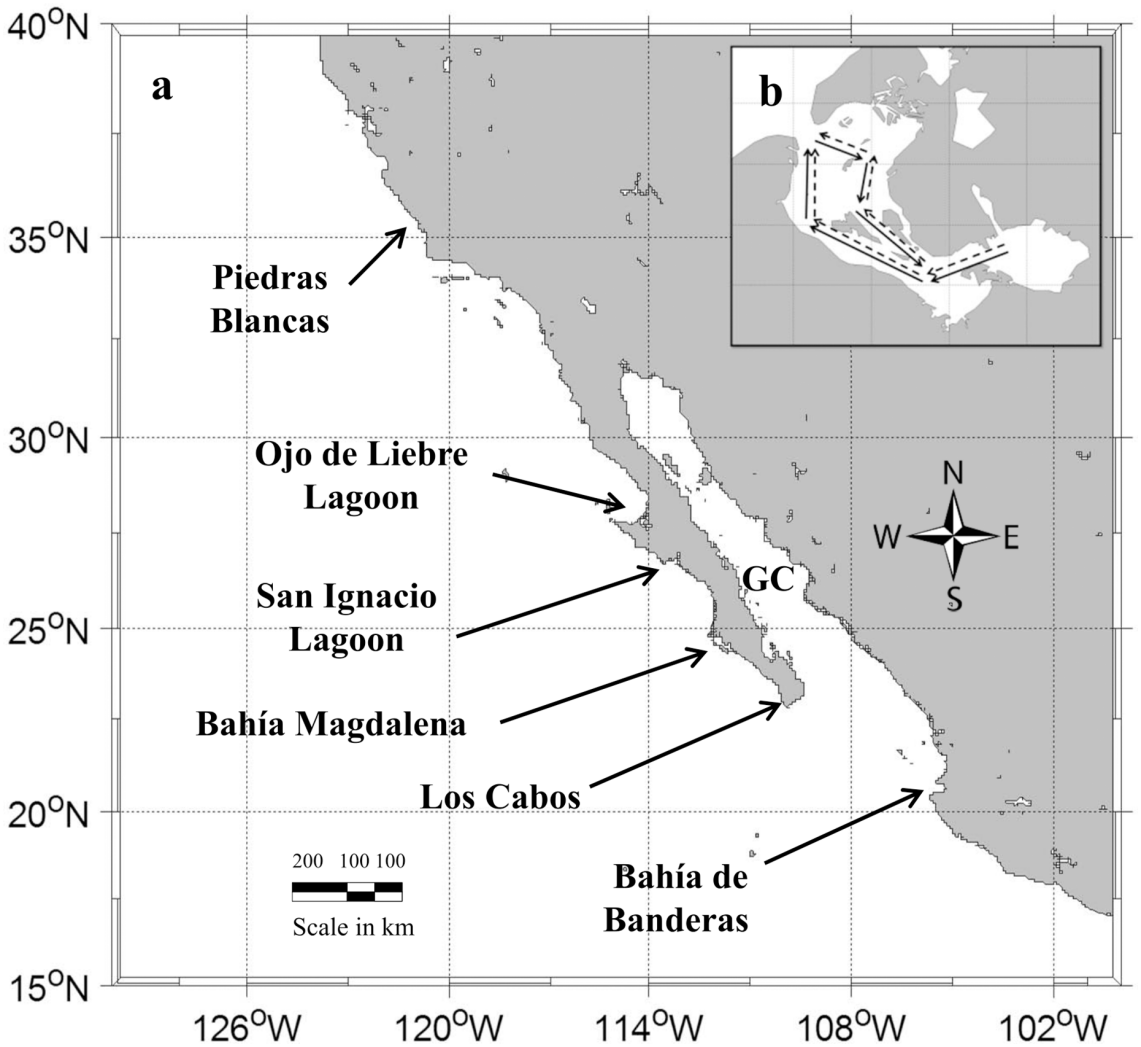
Study area. a) Breeding area details (GC: Gulf of California); b) the predetermined survey transect followed when counting gray whales in Ojo de Liebre Lagoon from 1980 to 2000 (solid line) and from 2000 to 2009 (dotted line).

The gray whale (*Eschrichtius robustus*) is distributed throughout coastal areas in the North Pacific. Two gray whale populations are currently recognized: the Western North Pacific population, comprising approximately 140 individuals [1, 2], and the Eastern North Pacific (ENP) population, comprising approximately 20,000 individuals [3]. During the summer and fall months, ENP gray whales are distributed throughout their feeding grounds in the Bering, Chukchi, and Arctic Seas where they feed mostly on benthic and epibenthic fauna [4]. At the end of the feeding season, ENP gray whales undertake an 8,000 km migration (16,000 kilometers round trip) southward to their winter breeding grounds. These winter aggregation areas are found in and around three subtropical lagoons along the Pacific Coast of Baja California, Mexico: the lagoon complex of Bahia Magdalena (BM) in the south, San Ignacio lagoon (SIL), and Ojo de Liebre lagoon (OLL) in the north. Additional whales are found in fewer numbers in other coastal bays along the Pacific shore of Baja California Peninsula, Mexico, and California, USA [5] (Fig 1).

The winter aggregation areas of ENP gray whales are located within the southern portion of the California Current System. The climate at this region fluctuates at inter-annual and inter-decadal scales related to the El Niño southern oscillation (ENSO) and the Pacific Decadal Oscillation [6–8]. These climate variations have strong physical and biological signals, including changes in ocean productivity [9, 10], the abundance and distribution of small pelagic fish populations and other fish species [11–14], and the distribution of cetaceans [15–19]. Seasonal variation is the dominant environmental phenomenon in gray whale summer feeding areas, with significant inter-annual and decadal variability related to large-scale climate patterns. A combination of factors appears to drive the attrition of the Arctic sea-ice pack, including flux of warm water into the Arctic Ocean, air temperatures and wind-driven advection of sea-ice [20–22].

It has been suggested that gray whales may serve as ecosystem sentinels, or indicators of environmental variability, due to their apparent sensitivity and response to environmental changes [23]. The effect of climate variability on gray whales occurs at different spatial and temporal scales, causing changes in their distribution, reproduction, survival, and migration timing [15, 24–27]. For example, fluctuations in the number of northbound migrating gray whale calves leaving the breeding grounds during the spring were positively correlated with the length of time that primary feeding habitat was free of seasonal ice during the previous summer [27]. Because OLL has the largest wintertime abundance of mother-calf pairs and mating gray whales [5], and if the number of calves born each year is influenced by the amount of time their mothers had to feed in the Bering Sea during the previous summer, there should be a correlation between the length of the open-water season on the primary feeding grounds during the previous summer and the number of MC pairs observed in the OLL. The validity of this relationship can be assessed using an analysis similar to Perryman et al. for northbound migrating calves [27], but comparing counts of MC pairs at OLL rather than numbers of calves migrating past the Pt. Piedras Blancas, CA.

Changes in annual counts of gray whale at BM and SIL suggest that the whales’ distribution and duration of stay in these areas is influenced by warm and cold ENSO events [25, 26]. If ENSO conditions influence the distribution of gray whales among the three coastal breeding lagoons during the winter months, a similar response should occur in OLL. The maximum counts of gray whales in February (the peak of the winter breeding season) in the three primary breeding lagoons of OLL, SIL, and SDCh (for BM) were compared with sea surface temperature anomalies and ENSO index to see if there are correlations with the whales’ distribution in the 3 lagoon areas during El Niño and La Nina conditions between 1997 and 2002. These dates were chosen because there were whale counts available for all three lagoon areas in those years, and were documented a strong El Niño and La Niña related temperature anomalies during these years.

## Materials and Methods

### 2.1 Assessment of inter-annual change in counts of MC pairs at OLL and the extent of seasonal Arctic sea-ice

A long-term dataset of gray whale survey counts conducted during the winter breeding season in the OLL, and during the spring northward migration past the Pt. Piedras Blancas (Fig 1) from 1980 to 2009 (Table 1, S1 Dataset) were analyzed for correlation with seasonal Arctic Sea-ice. There are several gaps in the survey time series for OLL, with the largest occurring from 1990 to 1995 (Table 2).

**Table 1:** Sources of mother-calf pairs data. Site: site where the survey was done (from north to south): Piedras blancas (PB), Ojo de Liebre Lagoon (OLL), San Ignacio Lagoon (SIL), Santo Domingo Channel (SDCh) north of Bahia Magdalena; year: years when the surveys were done; period: months of the year when the surveys were done; led by: person who led the surveys, VRB: El Vizcaino Biosphere Reserve; Source: reference where the data was taken.

Site	Years	Period	Led by	Sources
PB	1997–2000	Mar-Jun	Perryman W	[27]
	2000–2009	Mar-Jun	Perryman W	[30]
OLL	1980–1983	Feb	Fleischer L	[5]
	1985–2000	Feb	Sánchez J	[5]
	2001–2002	Feb	Urbán J	[5]
	2003–2009	Feb	Personnel VBR	This paper
SIL	1997–2002	Feb	Swartz S, Urbán J	[5]
SDCh	1997–2000	Feb	Péréz-Cortés H	[5]
	2001–2002	Feb	Urbán J	[5]

**Table 2:** Long-term dataset and basic statistic of gray whale survey counts conducted during the month of February at OLL. Mean: sum of the total number of whales counted during all the surveys conducted during February divided by the total number of surveys conducted in that month (total whales/total surveys); SD: standard deviation; Max: survey with the maximum number of mother-calf pairs counted; Min: survey with the minimum number of mother-calf pairs counted.

	Total	Number of Mother-calf pairs				
Year	Surveys	Mean	Mean+SD	Mean-SD	Max	Min
**1980**					557	
**1981**					525	
**1982**					553	
**1983**					463	
**1985**	2	487	507	467	502	473
**1987**	3	447	572	322	534	303
**1988**	6	74	90	58	95	54
**1989**	5	71	86	56	91	49
**1996**	6	422	487	357	512	360
**1997**	3	538	619	457	626	466
**1998**	1	530			530	530
**1999**	2	165	233	96	213	116
**2000**	4	229	260	197	256	196
**2001**	2	216	229	202	225	206
**2002**	4	424	484	364	475	361
**2003**	3	363	403	322	401	367
**2004**	3	697	863	531	889	601
**2005**	4	696	837	555	841	508
**2007**	2	247	270	224	263	231
**2008**	4	256	327	184	344	187
**2009**	4	303	343	263	335	245

All the surveys in OLL were conducted from 7-m boats powered by an outboard motor, with two observers, one on each side of the boat looking for whales and a third person recording the number of whales sighted by each observer. The surveys followed a standard survey transect (Fig 1b), and the whale counts were obtained using standardized observer protocols for consistency of survey effort that would allow for inter-annual comparisons of whale counts. During each survey, the boat followed the predetermined survey transect (Fig 1b) using visual landmarks and a hand-held GPS while traveling at a speed of 11 km/h. As gray whales typically travel at 6 km/h [28], this survey speed limited the opportunity for whales to move ahead of the boat and be counted more than once. At the same time, this speed was not fast enough to miss whales that were below the surface, given the typical dive time of 1.0 to 2.6 min [29]. To avoid double counts, the whales were recorded only when they cross an imaginary line perpendicular to the observers on each side of the boat. This survey method was first proposed by Swartz and Jones [29] for SIL and then was adapted for surveys in OLL and BM. It is presumed that all the whales in the area surveyed were observed and counted because: 1) although OLL is a large bay, the whales are confined to a few deep channels where the water is of sufficient depth, and the surveys followed these channels; and 2) both shorelines were visible at all times along the entire survey line. The surveys were aborted whenever the estimated sea state exceeded Beaufort 3 (winds greater than 18 km/h and consistent white caps). Counts of northward migrating gray whale calves at Pt. Piegras Blancas were conducted by shore-based observers [27, 30].

Prior to 2000, the surveys in OLL were performed using one boat. However, beginning in 2001, two survey boats were used, with each boat following synchronized and parallel transect lines (Fig 1b) to provide a better coverage of the lagoon, to reduce the time necessary to complete a survey and reduce the likelihood of significant changes in the whales’ distribution during a survey, and to further reduce the likelihood of double counting.

Data were grouped into single whales (adult whale without a calf) and MC pairs. Several surveys were conducted during the peak of the winter breeding season in February (Table 2). From these, the surveys with the highest number of mother-calf pairs recorded that month were used for the analysis (hereafter referred to a “maximum counts of MC pairs”). Counts from February are the best indicators of the annual mother-calf pairs present in the OLL lagoon because the peak of the breeding season occurs in February and maximum whale counts were obtained at that time [5]. Additionally, February was the best sampled month across all breeding seasons, with the most surveys conducted (Table 2).

The maximum count of MC pairs was used as an index of the trends in the number of calves born and the use of OLL as a breeding site in a given breeding season. The total calf estimate from the number of mother-calf pairs counted passing near shore at Piedras Blancas Light Station (Fig 1) during the spring northbound migration (Table 1) was used as an index of the number of calves born into the ENP gray whale population in a given year.

To assess whether changes in the inter-annual presence of MC pairs at OLL were related to environmental changes at their feeding areas between 1979 and 2009, the length of the open-water season at the Bering Sea was used as an index of sea ice extension at gray whale feeding areas during summer-fall months (Bering Sea sea-ice data was obtained from Brown and Arrigo [22], S1 Dataset). The length of the open-water season is defined as the number of days elapsed between the date of sea-ice retreat and the date of sea-ice advance. The date of sea-ice retreat was defined as the date when open water area in a specified region of interest rose above a given threshold: in the Bering Sea, this threshold was 90% of the total area. Similarly, the date of sea-ice advance was defined as the date when open-water area reduced by 90% [22].

To exclude the effects of variation in the survey effort and to verify that the number of MC pairs is a function of their abundance in the lagoon, the counts of mother-calf pairs during February were plotted and tested for correlation with the surveys effort (Spearman correlation with p < 0.05 as the significance level). To assess if any changes in mother-calf pairs at OLL are related with trends in the bulk of the population, the maximum counts of mother-calf pairs at OLL were plotted and tested for correlation with the “total calf estimate” from the Piedras Blancas surveys (Spearman correlation with p < 0.05 as the significance level). To assess if changes in mother-calf pairs at OLL are related with changes in their feeding areas in the Arctic reflected as changes in reproduction in the bulk of the population, the maximum counts of MC pairs at OLL during February and “total calf estimate” from Piedras Blancas were plotted and tested for correlation with the length of the open-water season of the previous feeding season at the Bering Sea (Spearman correlation with p < 0.05 as the significance level), as Perryman et al [27] was done and published.

### 2.2 Assessment of changes in the distribution of MC pairs at the three main breeding lagoons during contrasting ENSO conditions

Gray whale survey counts from the two southernmost breeding lagoons were compared with the OLL data (Table 1; S1 Dataset). These data were collected at SDCh (north of BM) and SIL (Fig 1). Surveys at these two sites were conducted following the same survey methodology described for OLL. In terms of area and overall numbers of gray whales, OLL is the most important breeding area, followed by SIL and BM, and the counts of MC pairs in these three lagoons represented more than 90% of the gray whales wintering along the Baja California coast [5]. Changes in the number of MC pairs residing in these lagoons in different years during the same survey dates was used as an indicator of changes in the whales’ winter distribution. The counts of MC pairs on similar dates were believed to be representative of the seasonal distribution of gray whales among the lagoons because lagoons are separated by at least 200 km (Fig 1), and minimize the likelyhood of the immediate exchange of animals between the lagoons.

The abundance of MC pairs for each breeding season was represented by the February survey that recorded the maximum number of MC pairs at each site (maximum counts). The “maximum count” of calves at each site was expressed as the percent of the “total calf estimate” from surveys at Piedras Blancas, and computed for each breeding season (% of calves at one lagoon = Maximum counts of mother calf pairs from that lagoon/total calf estimates from Piedras Blancas Light Station). Using the percent of the “total calf estimate” excludes the effects of changes in total calf numbers in the bulk of the population due to sea ice condition at their feeding ground and mortality during migrations. The percent of the total number of MC pairs that used each of the lagoons under different environmental condition allows detection of changes in their distribution that are related to ENSO effects. The data available for analysis were gray whale counts from 1997 to 2002 because these were the only years with surveys in the three lagoons and estimates of the total number of calves from Piedras Blancas Light Station.

To characterize the climate condition during these years, two environmental variables were obtained (S1 Dataset). First, monthly mean sea surface temperatures (SST) were extracted for a 2° x 2° squares located in front of the entrance of the three lagoons using the “Extended Reconstructed Sea Surface Temperature” data provided by the Earth System Research Laboratory (http://www.esrl.noaa.gov/psd/data/gridded/data.noaa.ersst.html). The long-term mean and seasonal signals of SST were calculated by fitting annual and semiannual harmonics to the 30-year monthly mean time series [31]. The SST anomalies (SSTAs) were then computed as residuals containing the low-frequency variability (inter-annual and decadal scales) after extracting the long-term mean and seasonal signals. Second, the monthly values of the Northern Oscillation Index (NOI) were downloaded from the Pacific Fisheries Environmental Laboratory (http://www.pfeg.noaa.gov/products/PFEL/modeled/indices/NOIx/noix.html). This index reflects the variability in the equatorial Pacific and extra-tropical tele-connections and represents a wide range of local and remote climate signals related to the ENSO inter-annual variability in the northeastern Pacific [32].

Although only whale data during the peak of the breeding season (February) were used in the analysis, the bi-monthly average (January-February) for the SSTA and NOI index for each breeding season were used for comparison because the photographic records suggested that mother-calf pairs stayed in the lagoon area up to 76 days with an average of 22 days at OLL, and 25 days at SIL [5]; for that reason mother-calf pairs sighted in February could be affected by ENSO conditions in January as well.

The bi-monthly (January-February) mean for each breeding season of the SSTA and NOI index were plotted and tested for correlation (Pearson correlation with p < 0.05 as the significance level) as a means of evaluating the relationship between the local SSTA variability at the breeding areas with NOI variability at wintertime. This analysis was done with a sufficiently long time (from 1980 to 2009) to ensure that the SSTA at the three sites have similar response to the ENSO pattern pointed out by the NOI index. Finally, the maximum counts of MC pairs and percentages they represented for each lagoon between 1997 and 2002 were then plotted with the NOI index values for January-February average conditions. This was tested with a lineal trend curves to evaluate the distribution shifts related to ENSO phenomena (Linear regression test with p < 0.05 as the significance level). In addition, a regression by log-log comparisons was made to evaluate possible relationships that are not linearly parallel.

## Results

### 3.1 Assessment of inter-annual change in counts of MC pairs at OLL and the extent of seasonal Arctic sea-ice

**Fig 2:**
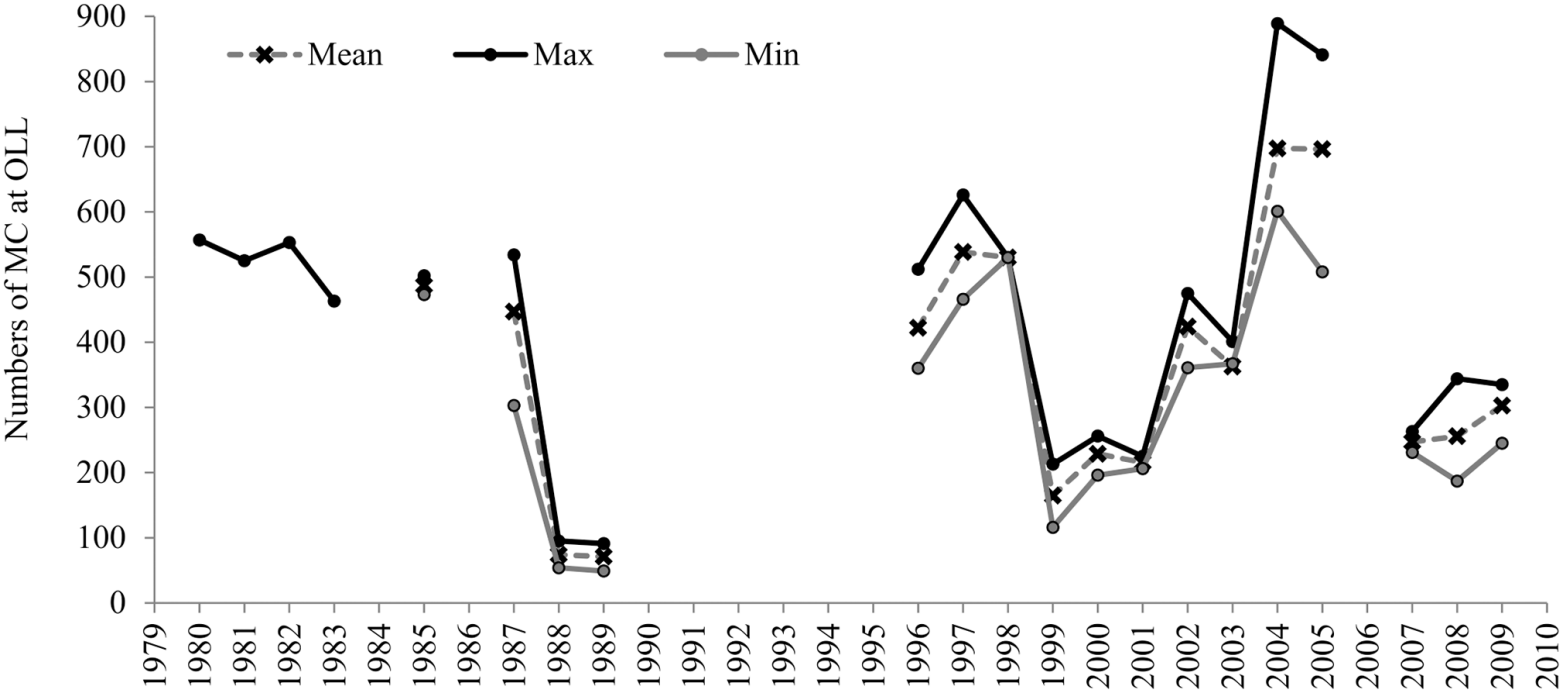
Mother-calf pairs counts at Ojo de Liebre Lagoon. Mean, maximum (Max) and minimum (Min) counts of mother-calf pairs (MC) at Ojo de Liebre Lagoon (OLL) during the month of February; Mean: sum of the total number of whales counted during all the surveys conducted during February divided by the total number of surveys conducted in that month (total whales/total surveys); Max: survey with the maximum number of MC pairs counted; Min: survey with the minimum number of MC pairs counted.

**Fig 3:**
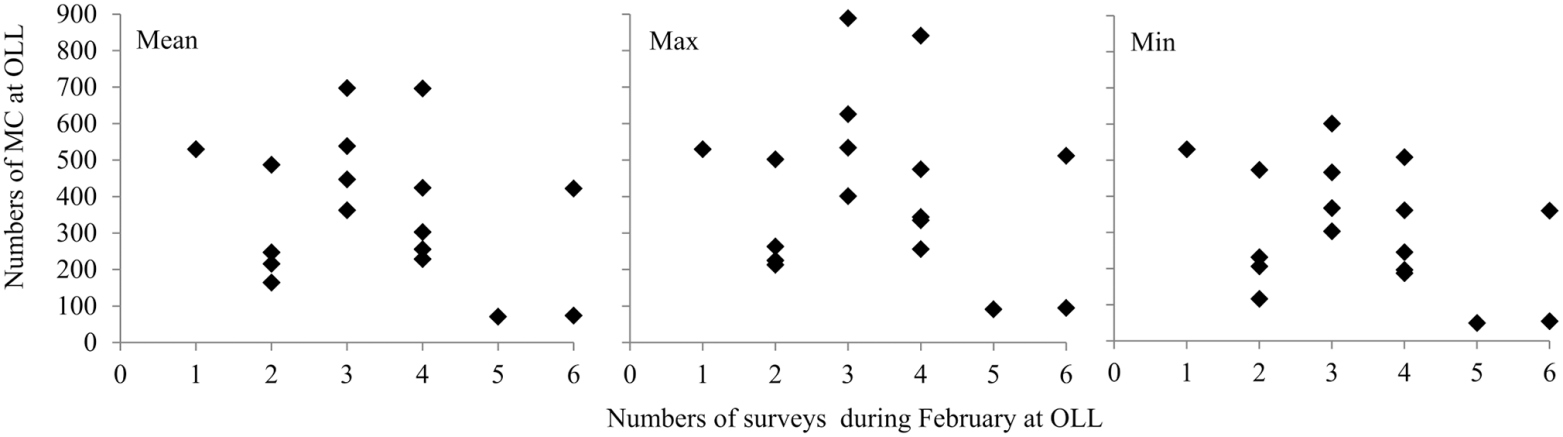
Mother-calf pairs counts vs survey effort at Ojo de Liebre Lagoon. Mean, maximum and minimum counts of mother-calf pairs (MC) at Ojo de Liebre Lagoon (OLL) during the month of February vs the total number of surveys conducted in that month; Mean: sum of the total number of whales counted during all the surveys conducted during February divided by the total number of surveys conducted in that month (total whales/total surveys); Max: survey with the maximum number of MC pairs counted; Min: survey with the minimum number of MC pairs counted.

**Fig 4:**
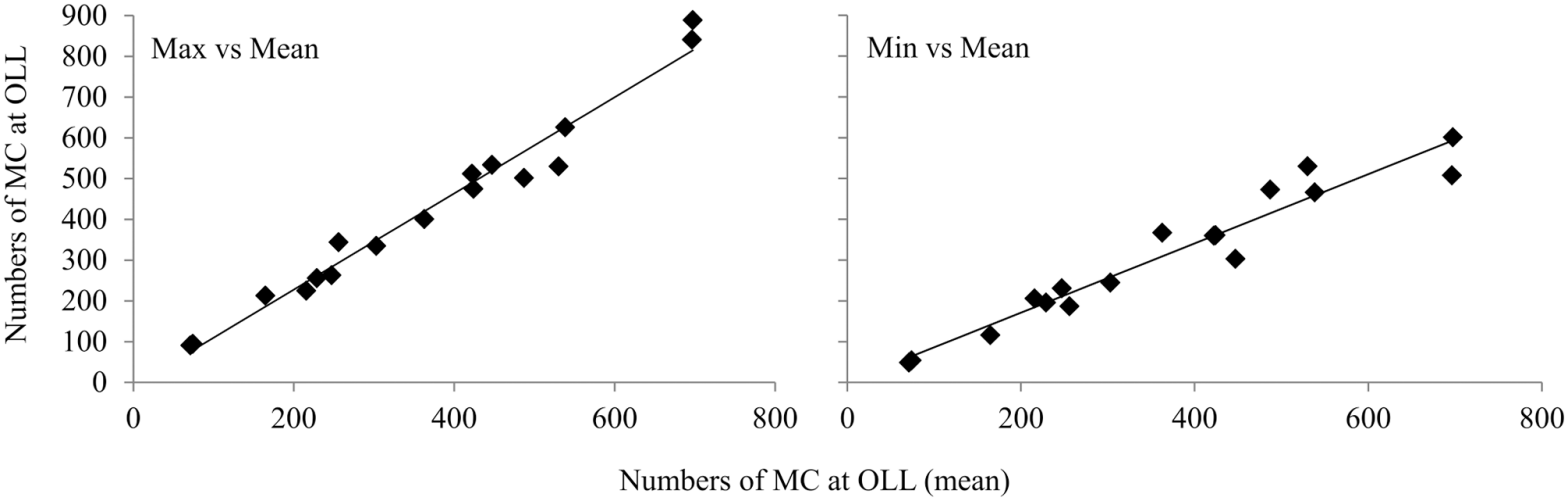
Mean mother-calf pairs counts vs maximum and minimum counts at Ojo de Liebre Lagoon. Mean, maximum and minimum counts of mother-calf pairs (MC) at Ojo de Liebre Lagoon (OLL) during the month of February; Mean: sum of the total number of whales counted during all the surveys conducted during February divided by the total number of surveys conducted in that month (total whales/total surveys); Max: survey with the maximum number of MC pairs counted; Min: survey with the minimum number of MC pairs counted.

On average, 400 (± 183 SD) MC pairs used OLL during the month of February between 1980 and 2009. The maximum whale counts of MC pairs showed pronounced inter-annual fluctuations, with maximum counts above of 800 MC pairs during the 2004–2005 breeding season and less than 100 pairs during 1989 breeding season (Fig 2). Although, there is no uniformity in the number of surveys conducted each year (Table 2), when the mean, maximum and minimum numbers of animals counted versus the survey effort are compared, the numbers of MC pairs counted are independent of the survey effort and represent a function of their abundance in the lagoon (Fig 3). This approximation suggests that the values of the maximum counts of MC pairs are representative of the average number of whales relative to the survey effort (Figs 3 and 4); suggesting these measures are equivalent. However, the maximum counts of MC pairs was used in the analysis because it provides an index of the total number of MC pairs that used the lagoon as breeding area, and because estimates of the maximum number of MC pairs counted were available only for 1980 to 1983.

**Fig 5:**
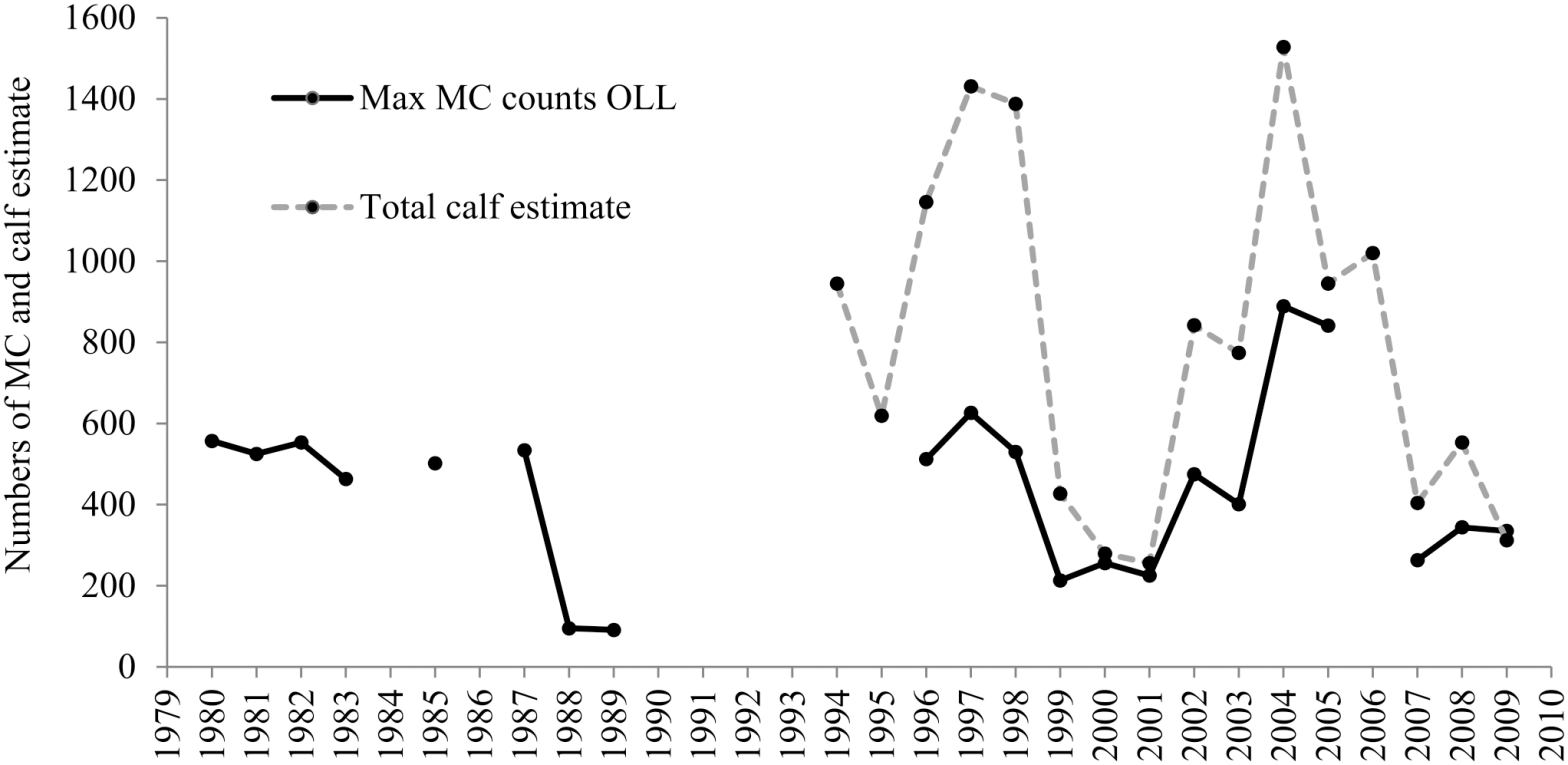
Temporal trends in mother-calf pairs at Ojo de Liebre Lagoon and total calf estimate. Temporal trends in maximum counts of mother-calf pairs (MC) at Ojo de Liebre Lagoon (OLL) during the month of February; and total calf estimate from Piedras Blancas nearshore surveys.

The maximum counts of MC pairs in OLL were strongly correlated with the total calf estimate from Piedras Blancas near shore surveys (*R*_*Spearman*_ = *0.90*, *p < 0*.*01*, *n = 13*, Fig 5). These relationships indicate that the numbers of MC pairs at OLL closely track reproductive trends of the bulk of gray whale population.

**Fig 6:**
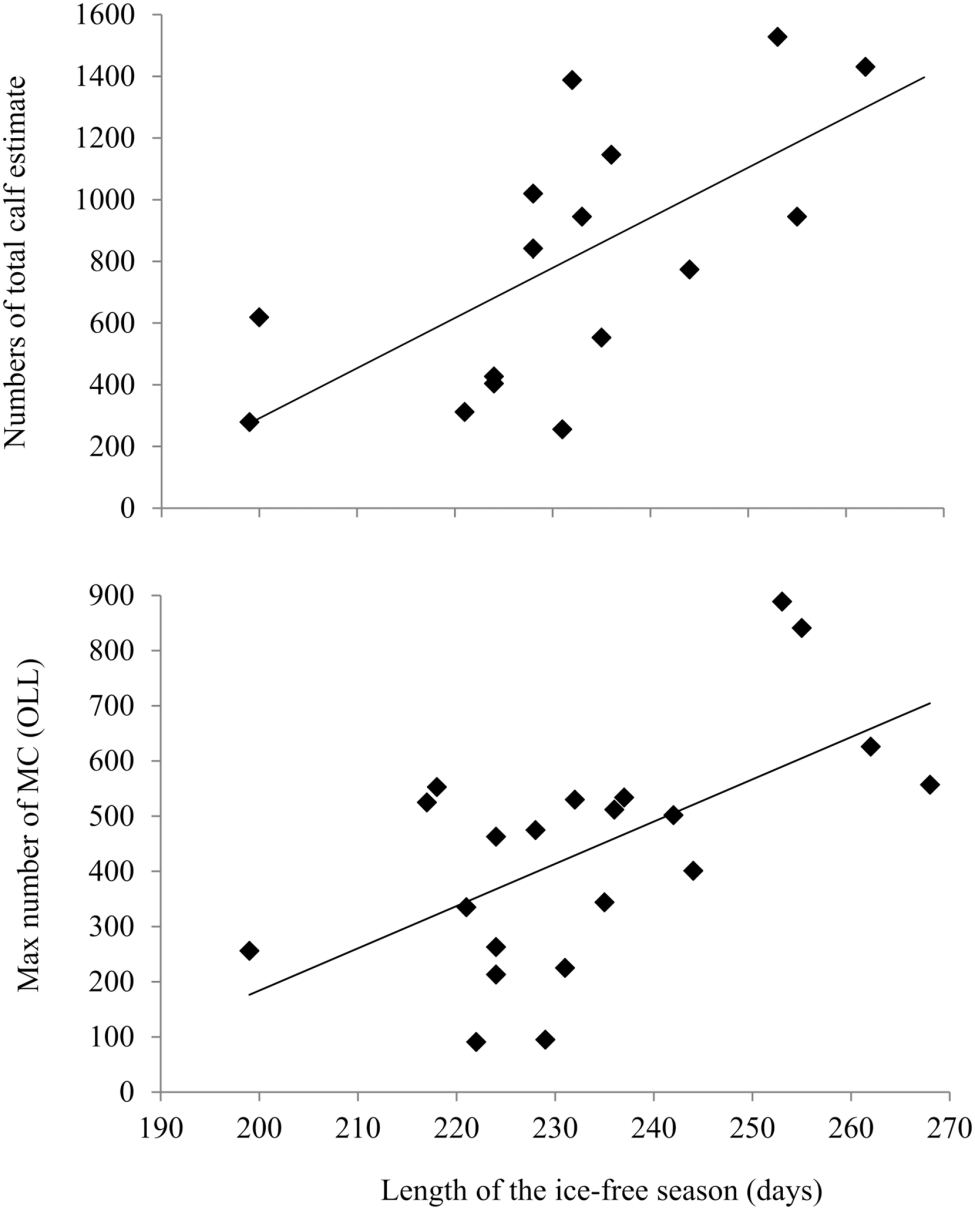
Mother-calf pairs vs previous summer conditions at the Bering sea: Upper panel. Total calf estimate from Piedras Blancas nearshore surveys vs length of the ice-free season of the previous feeding season at the Bering Sea; lower panel: maximum counts of mother-calf pairs (MC) at Ojo de Liebre Lagoon (OLL) during the month of February vs length of the ice-free season of the previous feeding season at the Bering Sea.

**Fig 7:**
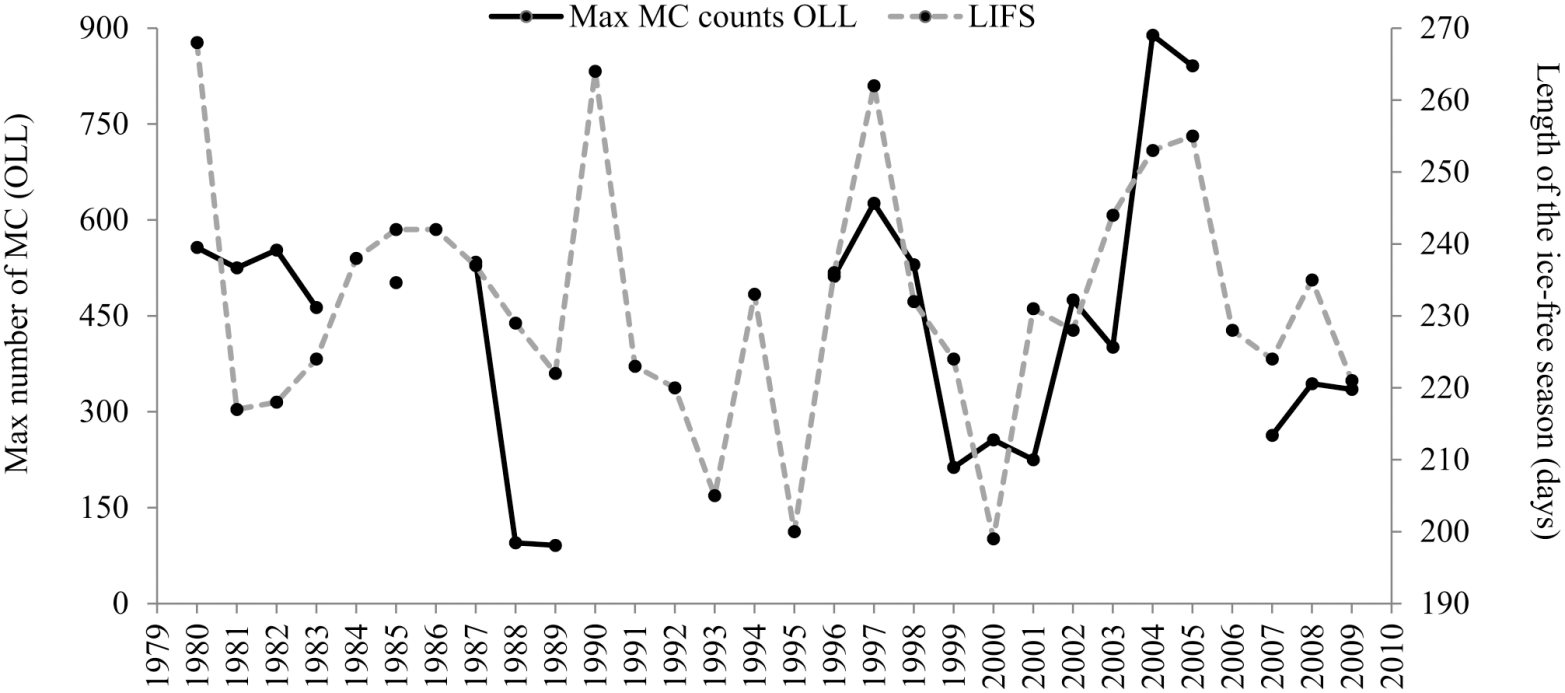
Temporal trends of mother-calf pairs and previous summer conditions at the Bering Sea. Maximum counts of mother-calf pairs (MC) at Ojo de Liebre Lagoon (OLL) during the month of February and length of the ice-free season (LIFS) of the previous feeding season at the Bering Sea.

The comparison of temporal trends between MC pairs at OLL and total calf estimate at Piedras Blancas showed a strong and positive relationship with the length of the open-water season of the previous feeding season at the Bering Sea (*R*_*Spearman*_ = *0.57*, *p = 0*.*01*, *n = 21*). This relationship indicates that the numbers of MC pairs counts at both sites respond to climate variability at their feeding grounds, with higher numbers of MC pairs occurring after an extended ice-free season, and lower numbers of MC pairs following a shorter ice-free season (Figs 6 and 7).

### 3.2 Assessment of changes in the distribution of MC pairs at the three main breeding lagoons during contrasting ENSO conditions

**Fig 8:**
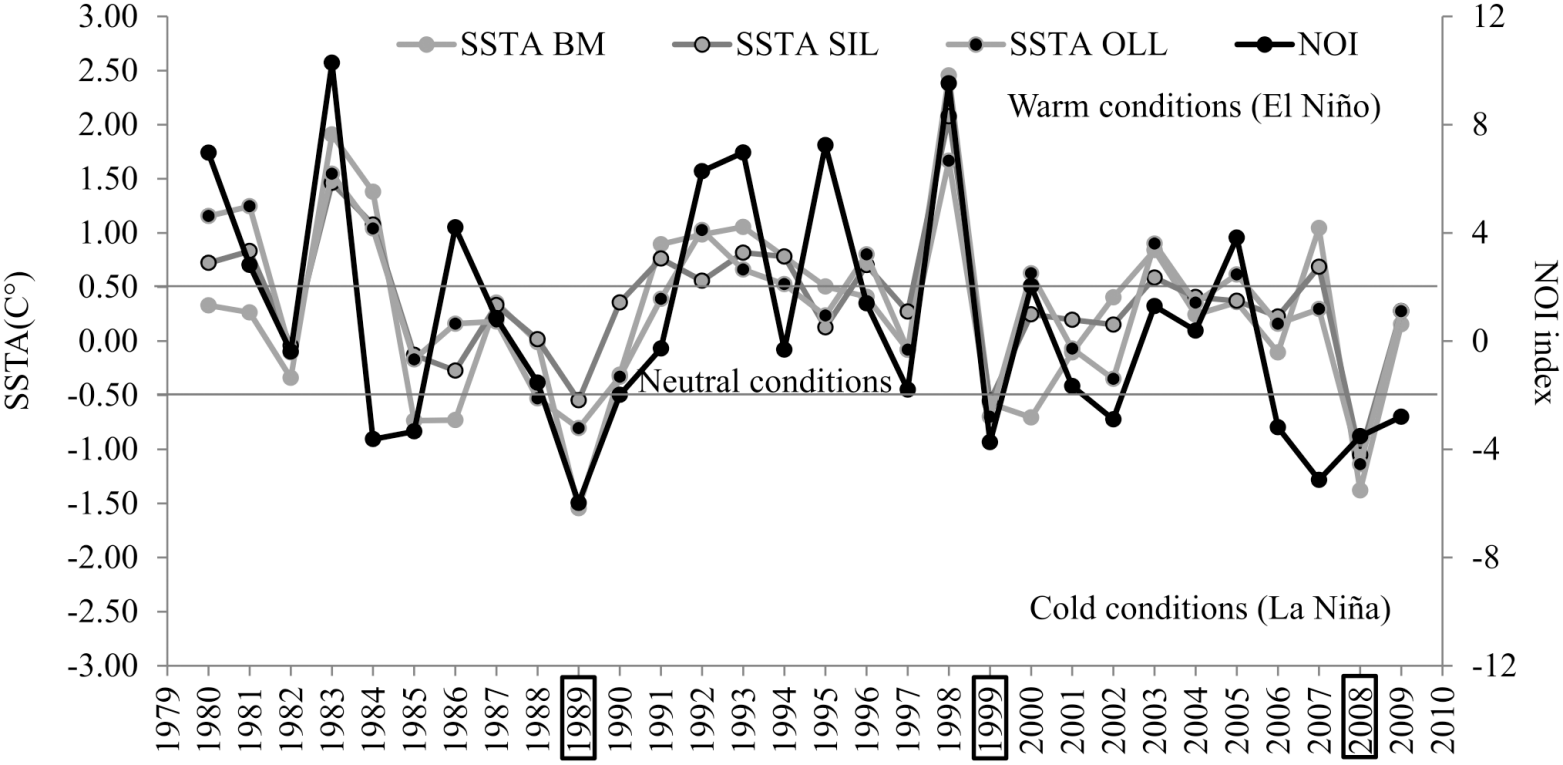
Climate variability at coastal areas in front of the breeding lagoons. mean (January-February) SST anomalies (SSTA) for Bahia Magdalena (BM), San Ignacio Lagoon (SIL) and Ojo de liebre Lagoon (OLL), and the ENSO index (NOI); the NOI index was multiplied by-1 for purposes of illustration; boxed years are reports of gray whale sightings in atypical southern locations [5, 26, 33].

The oceanic area adjacent to the three lagoons exhibit a strong inter-annual variability in terms of SST due to the latitudinal displacement of the sea surface isotherms, with warmer years (SSTA > 0.5°C) and colder years (SSTA < -0.5°C) (Fig 8). This inter-annual fluctuation in the SSTA closely followed the ENSO phenomenology represented by the NOI index (Table 3), with colder years during La Niña and warmer years during El Niño conditions (Fig 8).

**Table 3:** Correlation coefficient (R^2^) between sea surface temperature anomalies and ENSO index. Sea Surface Temperature Anomalies (SSTA) in the oceanic area at the entrance of (from south to north): Bahia Magdalena (BM), San Ignacio Lagoon (SIL) and Ojo de Liebre Lagoon (OLL); Northern Oscillation Index (NOI).

NOI vs	R^2^	p	N
**SSTA BM**	0.57	0.01	30
**SSTA SIL**	0.58	0.01	30
**SSTA OLL**	0.72	0.00	30

**Fig 9:**
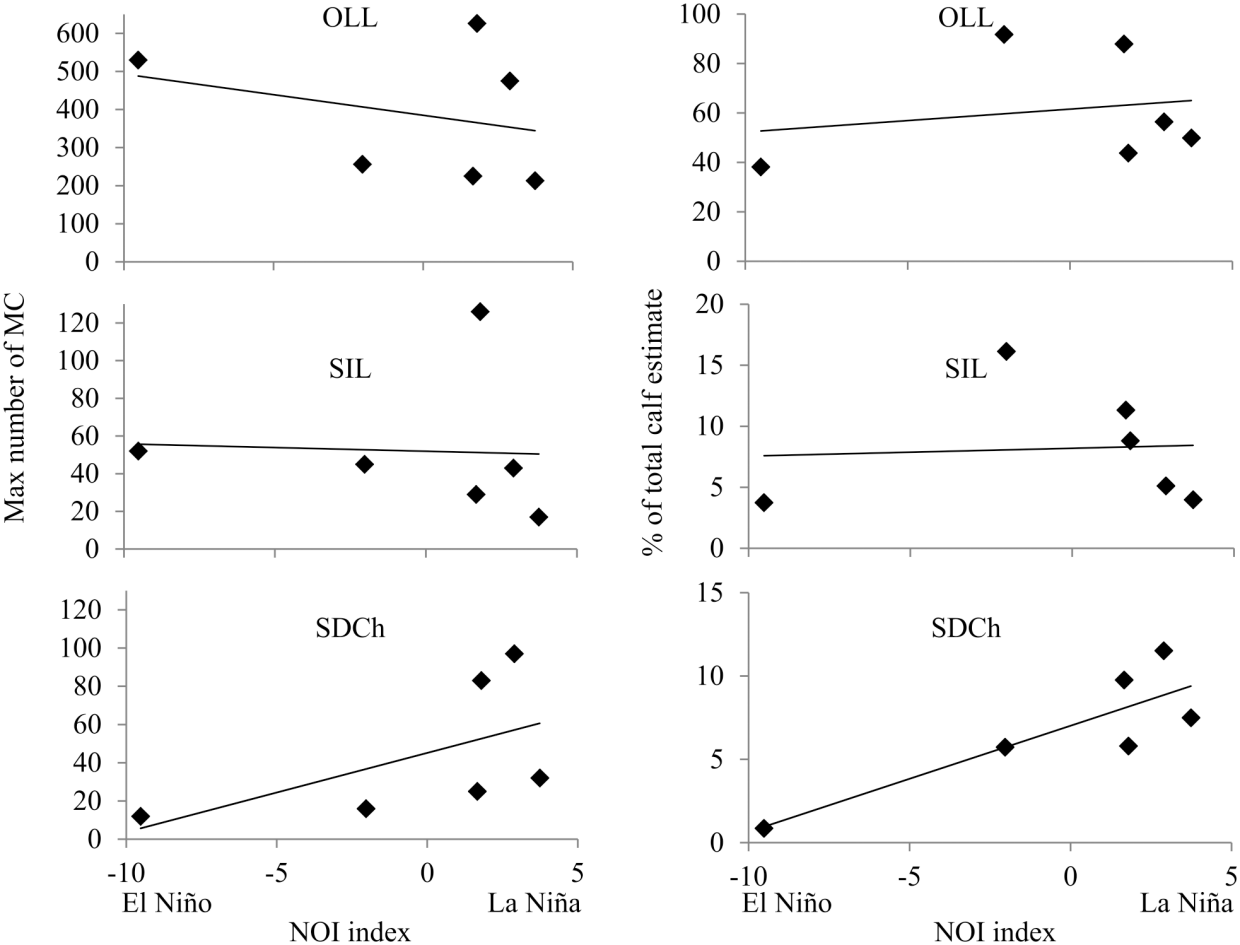
Mother-calf pairs distribution and ENSO conditions. Maximum counts of mother-calf pairs (MC) and the percent respect to the total calf estimate at Piedras Blancas for Santo Domingo Channel (SDCh) north of Bahía Magdalena, San Ignacio Lagoon (SIL), and Ojo de Liebre Lagoon (OLL) vs the Northern Oscillation Index (NOI) for the years 1997–2002.

The maximum counts of MC pairs in each lagoon area and their percent of the “total calf estimate” did not show any change related with the ENSO conditions at OLL and SIL between 1997 and 2002 (Fig 9). However, maximum counts of MC pairs and their percent of the total calf estimate showed a significant linear trend related with ENSO conditions in the southern-most breeding and aggregation area at SDCh north of BM (*R linear regression = 0*.*84*, *p = 0*.*03*, *n = 6;* Fig 9). This significant relationship with NOI index indicated that the number of MC pairs increased in SDCh during the 1999 La Niña conditions and decreased during the 1998 El Niño conditions. In addition, the higher presence of MC pairs in SDCh during 1999 La Niña conditions coincides with unusual whale sightings as far south as Los Cabos, in the Gulf of California, and further south into the Bahia de Banderas along the mainland coast [5, 26, 33]. These unusual southern whale sightings also occurred under cold La Niña conditions in 1989 and 2008 [5, 26, 33] (Fig 8).

## Discussion

### 4.1 Assessment of inter-annual change in counts of MC pairs at OLL and the extent of seasonal Arctic sea-ice

The presence of MC pairs in OLL showed pronounced inter-annual fluctuations (Fig 2), and the maximum counts of MC pairs at OLL were strongly correlated with total calf estimates from near shore surveys of northbound gray whale calves at Piedras Blancas (Fig 5). This strong correlation is due to OLL hosting the largest number of gray whales of all the gray whales’ winter breeding and aggregation areas [5], and having the largest number of gray whale calves each year.

The comparison of temporal trends between MC pairs counts at OLL and total calf estimate showed a strong and positive relationship with the length of the open-water season of the previous feeding season at the Bering Sea (Fig 6), suggesting that the counts at OLL and Piedras Blancas have the same phenological response to the extent of Arctic sea-ice. This indicates that the numbers of MC pairs observed at OLL are a reflection of the “total calf production” as measured at Piedras Blancas during a specific breeding season; with larger numbers of MC pairs observed following an extended ice-free season on their feeding grounds, thus resulting in higher calf production; and fewer numbers of MC pairs counted following a shorter ice-free season, thus resulting in lower calf production.

Summer feeding areas for gray whales are restricted to specific shallow water regions that are ice-free for only part of the year. Sea-ice dynamics and the large proportion of shallow continental shelf waters influence the primary productivity regime that supports the prey populations of gray whales on their Arctic feeding grounds [23]. Sea-ice loss creates additional open-water habitat for phytoplankton, and a longer open-water season that has been found to be significantly correlated with greater annual primary productivity [22]. Moreover, the relationship between annual primary productivity and the extent of the open-water season is strongly influenced by Pacific inflow from the Bering Strait: the Chukchi, Siberian, and Beaufort sectors [22], areas where gray whales feed on benthic and epibenthic fauna [4]. Each spring newly pregnant female whales are the first to return to these feeding grounds and they must store adequate energy reserves as body fat to survive the period of fasting during the upcoming winter migration, the birth and lactation for their calves [27].

The extent of sea-ice is also a function of wind direction. During years when winds were more northerly, sea-ice concentrations over the Bering Sea middle shelf sector are up to fourfold greater than in years when winds were more easterly. These inter-annual variations in the Bering Sea open-water season influences annual primary productivity significantly. For example, the open-water season was 50 days longer in 2003–2004 than in 1999, which increased Bering Sea annual primary productivity around 30% to 40%, respectively [22]. Related to this reduction in sea ice, gray whale calls were detected in the western Beaufort Sea over the winter of 2003 and 2004, presumably because the whales had winter-time access to ice-free Arctic areas [34]. The highest counts of MC pairs at OLL occurred during the breeding seasons following extended ice-free periods (2004–2005), with an increase of over 100% from the average counts of mothers-calf pairs observed the whole period (Fig 7). While, the lowest counts of MC pairs (1999–2001, 2007–2009) occurred after a sharp decline in open water season (1998–1999, 2006–2008; Fig 7).

The low counts of MC pairs during the breeding seasons of 1999–2001 correspond with low calf production and an unusual increase in gray whale mortality during those years. These population responses can be attributed to the combination and feedback of two events: the decreased of prey availability along their feeding areas due to the strong 1997–1998 El Niño event [24] and the subsequent reduction in the length of the open water season during the following 1998–2000 breeding seasons (Fig 7). These changes in the MC pairs counts can be attributed to poor nutritional condition of females due to environmental effects that shorten the feeding season at both temporal and spatial manner, that subsequently impact recruitment to the population [27].

When they are nutritionally stressed, gray whales display characteristic signs of emaciation in the form of a depression behind the blowhole, as well as visible vertebrae spines, protruding shoulder blades and ribs. This condition is seen in some whales following summers with high and persistent ice cover that limits access to their feeding grounds [24, 35]. In this regard, body condition has been demonstrated to be an indicator of fitness, potential survival and reproductive success in whales. For example, lipids in blubber are used as energetic support for reproduction in female right whales (*Eubalaena* spp.) and the marked fluctuations in right whale reproduction have a nutritional component due to changes in prey abundance [36, 37]. Similar observations were made with humpback whales (*Megaptera novaeangliae*) in the southern hemisphere, where the evidence suggested a strong relationship between sea ice extension at their feeding grounds and inter-annual variability in their body condition [38]. In turn, it has been demonstrated in mysticetes that pregnant females with poor body condition reduce their energetic investment in their fetus proportionately to their condition, most likely to help maintain a high survival probability [39].

### 4.2 Assessment of changes in the distribution of MC pairs at the three main breeding lagoons during contrasting ENSO conditions

The oceanic area adjacent to the lagoons exhibit a strong inter-annual variability in the SSTA associated with ENSO due to the latitudinal displacement of the sea surface isotherms, with warmer years (SSTA > 0.5°C) during El Niño conditions and colder years (SSTA < -0.5°C) during La Niña conditions (Fig 8). Latitudinal movements of pelagic fauna have been documented along the coast of Oregon, California, and Baja California in response to ENSO variability. A common response to El Niño warming is the pole-ward shift in distribution of many pelagic and benthic strong-swimming species, such as tuna, marlin, and billfish; medium and small-sized pelagic fish, such as mackerel, sardine and thread herring; as well as other species, such as barracuda, bass, pompano and moonfish. La Niña events have effects that are opposite to those of El Niño, although northern faunal intrusions into subtropical areas have seldom been reported [40]. In addition, ENSO impacts on prey availability have been documented for several marine mammal populations in the Northeast Pacific [41–44].

The maximum counts of MC pairs in each lagoon area and their percent of the “total calf estimate” did not show any change related with the ENSO conditions at OLL and SIL between 1997 and 2002 (Fig 9). However, there was an evident shift in whale counts and percent at SDCh in Bahia Magdalena (Fig 9), the southernmost breeding lagoon (Fig 1), with a decreased of MC pairs during the 1998 El Niño conditions and an increased during the 1999 La Niña conditions. In addition, the lower number of MC pairs during 1998 El Niño conditions coincides with higher counts of MC pairs in California coast, and it is possible that these MC pairs did not migrate as far south as Mexico that year [15]. While the increased number of MC pairs during 1999 coincides with unusual whale sightings in the northern Gulf of California and Bahia de Banderas [26, 33], these sightings appear to emphasize the extent of the gray whale southward distribution shifting during colder La Niña conditions. These distribution changes support the hypothesis of a northward shift in the distribution of MC pairs during the 1998 El Niño conditions and a southward shift during the 1999 La Niña conditions [25, 26], but evident only in the in the southern extreme of their wintering distribution.

It is known that newborn cetaceans are more vulnerable than adults to temperature changes [45, 46]. Previous studies proposed that gray and humpback whales shift their distribution to reduce newborn thermal-stress in their breeding areas [15, 47]. When compared with previous studies, the analysis presented here indicates that both species apparently shift their distribution to the Ecuador during cooler years (e.g., the 1989 and 1999 La Niña events; see Fig 8). These observations support the hypothesis that links the temperature at wintering areas to energetic strategies [48]. Research on heat flow and metabolic rate of gray whales in the laboratory and under field conditions conducted by Sumich [49] between 1976 and 1981 suggested that calves that are smaller or thinner than normal, or calves of small mothers with limited lipid reserves, derive energetic benefits from the relatively warm water conditions found in the breeding lagoons. The warmer temperatures functions to reduce body heat losses, and may be essential to the calves survival by allowing mothers to utilize less lipid reserves during periods of lactation, until substantial feeding is resumed sometime after leaving the lagoons [49]. Thus, the temperature at the wintering areas most likely constitutes a selective force driving whale migration, as proposed for humpback whales [47].

Additional evidence supporting the hypothesis of latitudinal change in whale distribution in response to ENSO variability includes: 1) the unusual reports of gray whales as far south as Los Cabos, and Bahia de Banderas, during anomalously cold years of 1989, 1999 and 2008 [5, 26, 33], representative of the increased extent of the southward shift in the whales’ distribution during these colder years (Fig 8); and 2) the lowest counts of MC pairs at OLL (1988–1989) occurred after a feeding season with shorter than average length of the open water season (Fig 7), and there were no records of mass mortality in the western coast of California Peninsula as occurred in 1999 and 2000 [50].

## Conclusions

The evidence suggest that the numbers of MC pairs at OLL are a reflection of the changes in the total number of calves born in the whole population, and these changes in calves’ numbers can be attributed to changes in nutritive condition of females due to temporal changes in the length of the open-water season at their feeding areas, with higher number of calves born after an extended ice-free season, and lower number of calves born after a shorter ice-free season. Our results agree with similar findings by other researchers about polar climate effects on gray whale abundance at their wintering areas. This may be useful for predicting numbers of MC pairs at OLL during the coming season using the sea ice conditions of the previous feeding season (http://pafc.arh.noaa.gov/ice.php).

ENSO-related variability in water temperature also influences the wintering distribution of gray whale MC pairs, especially in the southern extreme of their distribution during strong ENSO events, like the 1998 El Niño and 1999 La Niña. This evidence supports the hypothesis that gray whales prefer more southerly areas during years with cold sea temperature (La Niña) and more northerly areas during warmer sea temperatures (El Niño) to reduce thermal-stress and optimize energy utilization for newborn calves and their mothers. This shift in MC pairs could be anticipated with ENSO forecasts (http://www.cpc.ncep.noaa.gov/products/analysis_monitoring/enso_advisory/), and it could be useful to predict the numbers of MC pairs at SDCh, an area with intense tourism activity dedicated to whale watching.

Indeed, such predictions could be incorporated into the marine ecological program management that is currently being prepared by the Mexican government and the scientific community (http://www.semarnat.gob.mx/temas/ordenamientoecologico/bitacora/Paginas/pacifico_norte.aspx). Moreover, traits of gray whale ecology could be used to track biological consequences of climatic variation in a specific region and time frame under specific ecological conditions.

## Supporting Information

S1 Dataset Biological and Environmental data. (XLSX)
